# FEDRO: a software tool for the automatic discovery of candidate ORFs in plants with c →u RNA editing

**DOI:** 10.1186/s12859-019-2696-6

**Published:** 2019-04-18

**Authors:** Fabio Fassetti, Claudia Giallombardo, Ofelia Leone, Luigi Palopoli, Simona E. Rombo, Adolfo Saiardi

**Affiliations:** 1 DIMES, Università della Calabria, Via Pietro Bucci 41 C, Cosenza, Italy; 2 Department of Mathematics and Computer Science, Università degli Studi di Palermo, Via Archirafi 34, Palermo, Italy; 3 LMCB, MRC, Cell Biology Unit and Department of Developmental Biology, University College, London, UK

**Keywords:** RNA editing, Plants, ORFs generation

## Abstract

**Background:**

RNA editing is an important mechanism for gene expression in plants organelles. It alters the direct transfer of genetic information from DNA to proteins, due to the introduction of differences between RNAs and the corresponding coding DNA sequences. Software tools successful for the search of genes in other organisms not always are able to correctly perform this task in plants organellar genomes. Moreover, the available software tools predicting RNA editing events utilise algorithms that do not account for events which may generate a novel start codon.

**Results:**

We present Fedro, a Java software tool implementing a novel strategy to generate candidate Open Reading Frames (ORFs) resulting from Cytidine to Uridine (*c*→*u*) editing substitutions which occur in the mitochondrial genome (mtDNA) of a given input plant. The goal is to predict putative proteins of plants mitochondria that have not been yet annotated. In order to validate the generated ORFs, a screening is performed by checking for sequence similarity or presence in active transcripts of the same or similar organisms. We illustrate the functionalities of our framework on a model organism.

**Conclusions:**

The proposed tool may be used also on other organisms and genomes. Fedro is publicly available at http://math.unipa.it/rombo/FEDRO.

## Background

In mitochondria and chloroplasts of flowering plants the direct transfer of genetic information from DNA to proteins is corrupted by mechanisms that produce primary nucleotide sequences different from the original DNA sequences, by altering the structure of RNA transcripts. The most common among these mechanisms is post-transcriptional mRNA editing, consisting in enzymatic modification of nitrogenous bases, almost exclusively *c*→*u* transformation [1]. Most RNA editing events are found in the coding regions of mRNAs and usually at the first and second position of codons, so that the corresponding amino acid is often different from that specified by the unedited codon [2]. Editing can also create new start and stop codons [3, 4] and it can occur in introns [5] and in other non-translated regions [6]. The use of editing to generate *aug* start codons is well described for plants chloroplasts [7, 8], but there is evidence of it also in plants mithocondria [9]. This phenomenon may represent another level of regulatory control of gene expression. Indeed, the introduction of a translational start codon can make an mRNA accessible for protein synthesis [1]. Within this general context, in plant mitochondria RNA editing proves essential for gene expression. In many cases this mechanism completes the genomic information and it is important to the creation of a functional ORF [10].

The mechanism of RNA editing in plant mitochondria makes it harder studying the transfer of genetic information from DNA to proteins, due to the intervening differences occurring between RNAs and their coding DNA sequences. For this reason, common software tools for gene search helpful in finding canonical genes often fail short in discovering genes in plants and therefore some mitochondrial proteins may remain unknown. Accordingly, complete sequencing of mtDNA of many organisms allowed the identification of canonical genes, but much of the informational content of plant mitochondrial genomes remains still undiscovered. Therefore, finding plant mitochondrial proteins and understanding how they integrate into metabolic and signaling pathways, yet represents a major challenge in cell biology.

Here we present FEDRO, a Java software tool implementing a methodology based on the simulation of *c*→*u* RNA editing in the mitochondria of plants. The end result is the prediction of proteins which have been not yet discovered and annotated [11]. Indeed, plant mitochondria may use the editing mechanism on crucial sites, causing the generation of new, i.e., *edited*, start codons *aug* from *acg*. FEDRO is a three-steps approach that first generates a collection of potential ORFs, that cannot be detected by classical discovery techniques, based on the RNA editing simulation of edited starting codons. Then the ORFs which are already known to be encoded as proteins in the input organism are filtered out. The final step is a comparison of the remaining sequences against the BLAST database according to the programs described in [12], in order to select only those potential ORFs which present high sequence similarity with proteins in other organisms, or with transcripts in the same or different organisms.

We illustrate the functionalities of FEDRO on the mtDNA of *Oryza sativa*, where previous studies systematically identified mRNA editing sites of known ORFs (e.g., [13]) but they did not investigate the possible generation of novel ORFs by the editing of the first codon. FEDRO allows to take into account this aspect, leading to the prediction of novel putative ORFs encrypted via *c*→*u* editing.

## Implementation

The proposed system aims to automatically simulate the editing mechanisms possibly causing the presence of proteins that are not imputable to ORF sequences obtained by traditional methods (e.g., ORF FINDER [14], STARORF [15]). This is rather meaningful in plants, as already discussed in the Introduction, since mtDNA editing mechanisms can involve nucleotide triplets leading to start and stop codons. In particular, we aim to generate putative proteins not yet discovered in a given input organism. The by far most frequent nucleotide substitution caused by editing is *c*→*u* at the RNA level, that is, *c*→*t* if we refer to mtDNA. Therefore we consider only this type of nucleotide substitution in our analysis.

**Fig. 1:**
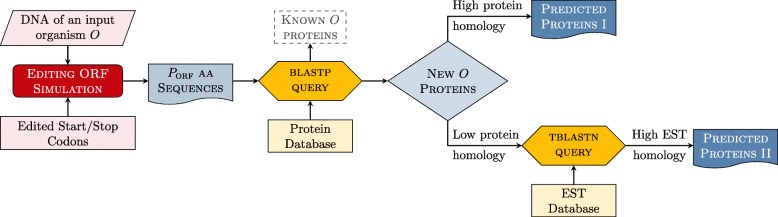
The protein prediction method based on editing simulation
- *Filtering*: comparison of the extracted ORFs with proteins already known in the same organism.
- *Selection*: selection of the non-filtered ORFs that show high homology with known proteins in other orgnanisms, and search of known transcripts in the remaining ORFs.

In particular, the proposed framework FEDRO consists of three main steps, graphically illustrated in Fig. 1:

- *Extraction*: ORFs extraction by editing simulation.

**Fig. 2:**
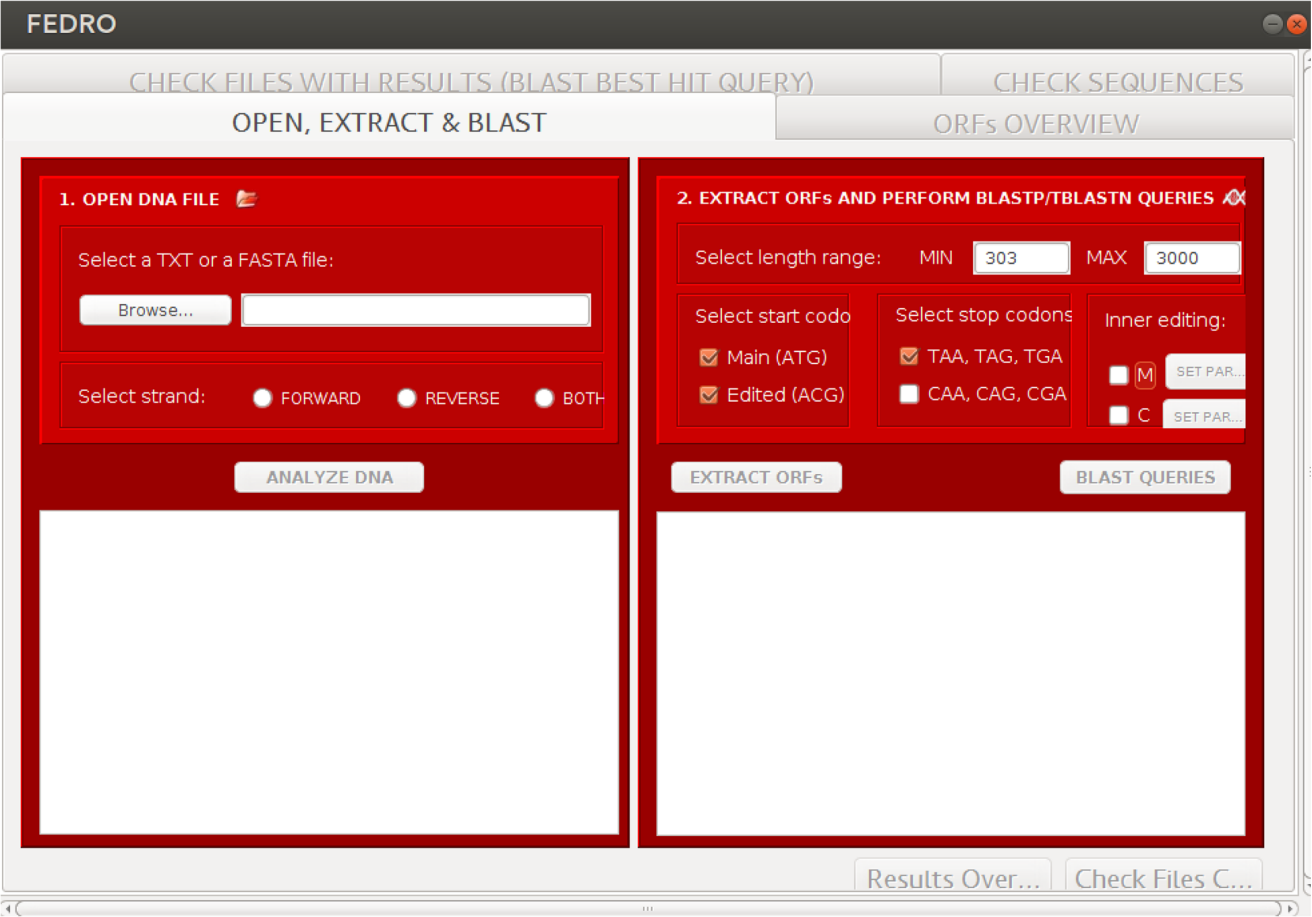
A screenshot of the main window of FEDRO

Figure 2 shows a screenshot of the main window of FEDRO. In the following we describe in detail each of the involved steps.

### Extraction

This phase is devoted to extract candidate ORFs from the input mtDNA sequence. We aim to extract both ORFs identified by standard start/stop codons and ORFs generated by the *c*→*t* editing mechanism using simulation. It is worth pointing out that editing events on the start/stop codons may affect the entire process of ORFs extraction. That is, although FEDRO returns also ORFs identified by standard start/stop codons, they could be different from those returned by ORF FINDER [14] or STARORF [15], due to different cuts of the input mtDNA sequence.

**INPUT:**

- the mtDNA sequence of an organism to be analyzed;
- user-defined threshold *θ*_*len*_: the minimum length of extracted ORFs, equal to 300 by default;
- considering edited start codons (*yes*/*no*), set to *yes* by default;
- considering edited stop codons (*yes*/*no*), set to *no* by default;

**METHOD:** The simplest way to find genes in a genome is that of scanning the input nucleotide sequence by considering all its three possible reading frames, then searching for nucleotide triplets corresponding to *start codons* and *stop codons* on a frame, and cutting the sub-sequences comprised between a start and a stop codon, that are the Open Reading Frame sequences. An ORF sequence is considered to be a potential protein encoding segments if its length is at least 300 nucleotides. In particular, there exist one start codon, that is, *atg* and three stop codons, that are *tag,tga* and *taa*. Although ORF sequences can be easily searched for in a genomic sequence by using existing software tools, such as for example ORF FINDER [14] and STARORF [15], these tools do not take into account the role of possible editing processes which could have modified the involved sequences.

Our system simultaneously looks for *atg* start codons and *acg* edited start codons. Next, it looks for a stop codon and extracts the ORF if and only if its length amounts at least at the minimum user-defined threshold *θ*_*len*_. Note that, since the stop codon does not have other possible interpretations, it cannot be ignored in order to find largest ORFs. All that given, two issues deserve attention:

1. that possible further start codons between the current start codon and the stop codon are ignored;
2. that edited stop codons are not taken into account.

The first issue is related to the fact that a start codon can be also be interpreted as amino-acid and using a preceding start codon just generates larger ORFs. Let $\overrightarrow {\delta }$ be the position where a start codon begins and let $\overleftarrow {\delta }$ be the position where a stop codon begins. Assume that the number of basis between $\overrightarrow {\delta }$ and $\overleftarrow {\delta }$ is larger than *θ*_*len*_. Consider now a possible further start codon beginning in a position *δ* between $\overrightarrow {\delta }$ and $\overleftarrow {\delta }$. Taking *δ* as the start codon beginning position leads to consider ORF comprised between *δ* and $\overleftarrow {\delta }$, which is strictly included in the ORF comprised between $\overrightarrow {\delta }$ and $\overleftarrow {\delta }$. Thus, ignoring intermediate start codons, like the one beginning at *δ*, implies to extract larger ORFs. As shown in the experiments, this does not lead the system to be less accurate.

The second issue is related to the fact that a stop codon does not have other possible interpretations and then each stop codon has to be considered to extract an ORF. Considering editing codons enlarges the set of stop codons to be taken into accounts and thus, leads to the generation of smaller ORFs.

**OUTPUT:** The output of this phase is a set of candidate ORFs, translated into amino-acid sequences.

### Filtering

Since the end goal of our system is to support the discovery of possible novel proteins, the goal of this phase is to filter out from the candidate ORF set, that set of ORFs which is already known to be encoded as proteins in the input organism.

**INPUT:** The set of candidate ORFs as returned by the extraction phase.

**METHOD:** To perform this operation, the system queries the protein database by exploiting the web services offered by the BLAST framework and, in particular, the system uses the blastp software to measure the similarity between a candidate and the known proteins, such that two proteins are considered similar if the resulting bitscore is larger than or equal to 200. From the analysis of the returned similarity, three cases can arise:

1. the ORF is similar to a protein already known in the input organism;
2. the ORF is similar to a protein already known in another organism;
3. the ORF is not similar to any already known protein.

Thus, this phase partitions the input set of ORFs in three sets:

- ORFs encoding proteins already known in the input organism (*to-drop* set);
- ORFs encoding proteins already known in other organisms;
- ORFs non-encoding known proteins.

**OUTPUT:** This phase outputs the two sets b) and c) of non-dropped ORFs.

### Selection

The goal of this phase is to select the set of ORFs to be returned for further analysis by domain experts.

**INPUT:** Two sets of candidate ORFs.

**METHOD:** The system returns the set of ORFs encoding proteins already known in other organisms. These ORFs are potentially useful for the prediction of putative proteins in the organism under analysis.

Next, the system takes into account the set of ORFs non-encoding known proteins. New proteins could belong to this set. In order to select promising candidates, the system queries the EST database by exploiting, again, the web services offered by the BLAST framework and, in particular, the system uses the tblastn software. The goal is to look for the presence of transcripts that may be indicative for a transcription of the ORFs sequence in a novel protein.

**OUTPUT:** Two sets of ORFs to be analyzed by domain experts.

## Results

To provide example of application, we show the results returned by FEDRO on the mtRNA of the model plant *Oryza sativa* (version *BA*000029.3).

As a first test, we extract all the possible ORF sequences from the considered mtDNA by considering only non-edited start and stop codons (with the constraint of a length equal to or larger than 300), as it is returned by a standard tool for ORFs extraction. Then, we do the same, but by applying FEDRO on the mtDNA of the input organism.

The result is that FEDRO generates standard ORFs which are not affected by editing on the start/stop codons, sometimes as subsequences or intersections with standardly observed ORFs. Most importantly, 43 out of 213, that is, 20% of the candidate ORFs returned by FEDRO cannot be extracted by standard tools, such as ORF FINDER [14] or STARORF [15], which do not consider editing events on the start/stop codons.

The second type of analysis aims at understanding if these putative ORFs result to be significant after the validation step, that is, they present high sequence similarity with known proteins or transcripts. In such a case, they can be put in a final catalogue of predictions to be provided to biologists for further lab inspections. In particular, Tables 1 and 2 show the results of the blastp queries for protein sequence similarity search. Results are sorted with respect to the BLAST bitscore values (also other BLAST scores can be taken into account analogously, such as e-value, percentage of coverage and percentage of identity; we omit them since the corresponding results do not present significant differences with respect to those presented here). The column ORF TYPE highlights if the corresponding ORF is contained in, or intercepts, another ORF returned by a standard tool (sub, int, respectively), or if it is not returned by a standard tool (new). Tables 3 and 4 are analogous to the previous ones, but they contain the putative ORFs resulting from tblastn query for EST sequence similarity search.

**Table 1:** Putative ORFs resulting from Blastp query for protein sequence similarity search obtained for *O. sativa*

seq	start	stop	strand	start	orf	orf	blastp	similarity found
nr.	type	type	type	codon	length	type	bitscore	organism
1	Main	Edited	fwd	32014	393	Sub	266	*O. nivara*
2	Main	Edited	fwd	45130	360	Sub	230	*Z. mays subsp. mays*
3	Main	Edited	fwd	322576	339	Sub	226	*O. sativa Indica Group*
4	Main	Edited	fwd	323041	348	Sub	223	*O. sativa Indica Group*
5	Main	Edited	fwd	334132	309	Sub	208	*O. sativa Indica Group*
6	Edited	Edited	rev	414167	327	New	205	*G. hirsutum*
7	Edited	Edited	fwd	372740	408	New	203	*Z. mays subsp. mays*
8	Edited	Edited	fwd	332693	315	Sub	200	*L. perenne*

Best hit bitscore and organism where the significant sequence similarity has been found are reported for each ORF. This table contains sequence similarity results corresponding to BLAST bitscore ≥ 200

**Table 2:** Putative ORFs resulting from Blastp query for protein sequence similarity search obtained for *O. sativa*

seq	start	stop	strand	start	orf	orf	blastp	similarity found
nr.	type	type	type	codon	length	type	bitscore	organism
1	Edited	Edited	fwd	283844	330	New	197	*V. faba*
2	Edited	Edited	rev	315377	330	New	195	*A. duranensis*
3	Edited	Main	rev	314493	342	New	194	*N. tabacum*
4	Edited	Edited	rev	461810	324	New	193	*T. aestivum*
5	Main	Edited	fwd	391725	327	Sub	187	*S. bicolor*
6	Main	Edited	fwd	361601	354	Sub	182	*T. aestivum*
7	Main	Edited	fwd	394888	360	Sub	179	*A. alpina*
8	Edited	Edited	rev	240111	321	Int	177	*C. cajan*
9	Edited	Main	fwd	331900	444	Sub	176	*S. italica*
10	Main	Edited	rev	98164	387	Sub	164	*P. edulis*
11	Edited	Edited	rev	424636	315	Int	163	*F. rimosivaginus*
12	Edited	Edited	rev	404262	363	New	156	*F. rimosivaginus*
13	Edited	Main	fwd	316040	309	New	150	*C. card. var. scolymus*
14	Edited	Main	fwd	142093	312	New	149	*S. bicolor*
15	Main	Edited	fwd	393830	339	Sub	147	*Z. mays subsp. mays*
16	Main	Edited	fwd	367055	306	Sub	130	*V. vinifera*
17	Edited	Edited	fwd	364454	327	New	129	*Z. mays subsp. mays*
18	Main	Edited	rev	260161	324	Sub	113	*T. subterraneum*
19	Edited	Main	rev	201474	348	New	113	*B. napus*
20	Edited	Main	fwd	232846	339	New	103	*A. thaliana*
21	Edited	Main	rev	232370	387	New	88	*G. raimondii*

Best hit bitscore and organism where the significant sequence similarity has been found are reported for each ORF. This table contains sequence similarity results corresponding to BLAST bitscore in the range 80−200

**Table 3:** Putative ORFs resulting from Tblastn query for EST sequence similarity search obtained for *O. sativa*FEDRO ORFs

seq	start	stop	strand	start	orf	orf	blastp	similarity found
nr.	type	type	type	codon	length	type	bitscore	organism
1	Main	Edited	fwd	50237	378	sub	216, 221	*O. officinalis*

Best hit bitscore and organism where the significant sequence similarity has been found are reported for each ORF. This table contains sequence similarity results corresponding to BLAST bitscore ≥ 200

**Table 4:** Putative ORFs resulting from Tblastn query for EST sequence similarity search obtained for *O. sativa*FEDRO ORFs

seq	start	stop	strand	start	orf	orf	blastp	similarity found
nr.	type	type	type	codon	length	type	bitscore	organism
2	Edited	Main	rev	105822	306	New	195	*O. longistaminata*
3	Main	Edited	rev	444521	354	Sub	194	*O. sativa Japonica Group*
4	Edited	Edited	rev	444885	411	New	194	*O. sativa*
5	Edited	Main	rev	55809	378	Sub	179	*O. punctata*
6	Main	Main	fwd	250268	342	Sub	171	*B. oldhamii*
7	Edited	Edited	fwd	407800	318	New	167	*P. virgatum*
8	Edited	Edited	fwd	354085	357	New	159	*O. longistaminata*
9	Edited	Edited	fwd	362648	315	New	145	*F. pratensis*
10	Edited	Edited	rev	449361	327	New	142	*T. cacao*
11	Edited	Edited	rev	295218	324	New	114	*V. vinifera*
12	Edited	Main	rev	207984	306	New	112	*O. sativa Japonica Group*
13	Edited	Main	fwd	385977	318	New	91	*S. officinarum*
14	Edited	Main	rev	248349	318	New	89	*O. sativa Indica Group*
15	Edited	Edited	fwd	203320	375	New	85	*T. cacao*

Best hit bitscore and organism where the significant sequence similarity has been found are reported for each ORF. This table contains sequence similarity results corresponding to BLAST bitscore in the range 80−200

The data displayed in the abovementioned tables show that FEDRO is able to successfully generate novel putative ORFs. They constitute a useful catalogue for the biologist in order to identify interesting sequences which may be associated to coding regions, but which cannot be recognized by standard ORFs extraction due to the presence of editing that modifies the ORF sequence start/stop codons.

## Conclusions

We propose FEDRO, a Java software tool implementing a novel strategy to generate candidate ORFs resulting from *c*→*u* editing substitutions which occur in the mitochondrial genome (mtDNA) of a given input plant. This is useful in order to predict putative proteins of plants mitochondria that have not been yet annotated.

We applied FEDRO on the mtDNA of *Oryza sativa*, suggesting a set of 45 novel putative ORFs to be verified by experts.

FEDRO may be usefully applied to single out informative subsequences also in other, less studied, organisms. With the catalogue of novel putative ORFs in hand, the biologist can perform further analysis and experiments in laboratory to discover the presence of novel proteins in plants, where the editing mechanisms alter the structure of RNA transcripts.

As our future work, we plan to apply our method on the chloroplast genome of plants, where the *c*→*u* editing also occurs. Moreover, we are working on the design of a methodology aiming at identifying possible coding exons of trans-spliced genes in the predicted ORFs. We will also investigate the chance to add alternative initial codons ORF [16] as a significant extra feature of the proposed system. Finally, another interesting direction of investigation regards a context-based analysis of the editing sites, which are already known and annotated for many model organisms. To this aim, both sequence motifs or *k*-mers based analysis may be considered (see, for example, [17, 18] and [19, 20], respectively) and approaches able to characterize anomalous contexts [21, 22].

## Availability and requirements

FEDRO is publicly available at http://math.unipa.it/rombo/FEDRO. As specified in the ’readme.txt’ file, the two files ’FEDRO.jar’ and ’data.properties’ have to be included in the same folder. Moreover, the blastp and tblastn executable files have to be provided, and the ’executable’ field of the ’data.properties’ file has to be updated with their paths.

## Abbreviations

BLAST: Basic local alignment search tool
EST: Expressed sequence tags
mtDNA: mitochondrial DNA
ORF: Open reading frames
